# Neocentromeres Come of Age

**DOI:** 10.1371/journal.pgen.1000370

**Published:** 2009-03-06

**Authors:** Owen J. Marshall, K. H. Andy Choo

**Affiliations:** 1 Chromosome and Chromatin Research, Murdoch Childrens Research Institute, Parkville, Victoria, Australia; 2 Department of Paediatrics, University of Melbourne, Parkville, Victoria, Australia; The University of North Carolina at Chapel Hill, United States of America

Sixteen years ago, the discovery of a newly formed, ectopic centromere in a human [1],[2] was a turning point for centromere research. Whereas previously centromeres had been thought of as immovable and unchanging, embedded in vast tracts of tandemly repeated DNA, this new centromere—or neocentromere—lacked any characteristic centromeric DNA sequences and had formed in a gene-rich area of the genome. Essentially, a fully functional centromere had spontaneously arisen where no centromere had any right to be, complete with all the necessary centromere proteins and epigenetic marks required for the creation of a complex DNA/protein structure. Neocentromere formation remains one of the most astonishing examples of epigenetic change within the genome.

Since this discovery, neocentromeres (not to be confused with the “classical” facultative neocentromeres, which were originally described in maize (reviewed in [3]) have been shown to be a means of centromere repositioning during karyotype evolution and speciation in vertebrates, with evidence suggesting a similar role in plants (for review, see [4]). Clearly, there is an evolutionary advantage in being able to form new centromeres, and this process has been conserved. However, an understanding of the mechanisms of neocentromere formation remains elusive.

**Figure 1:**
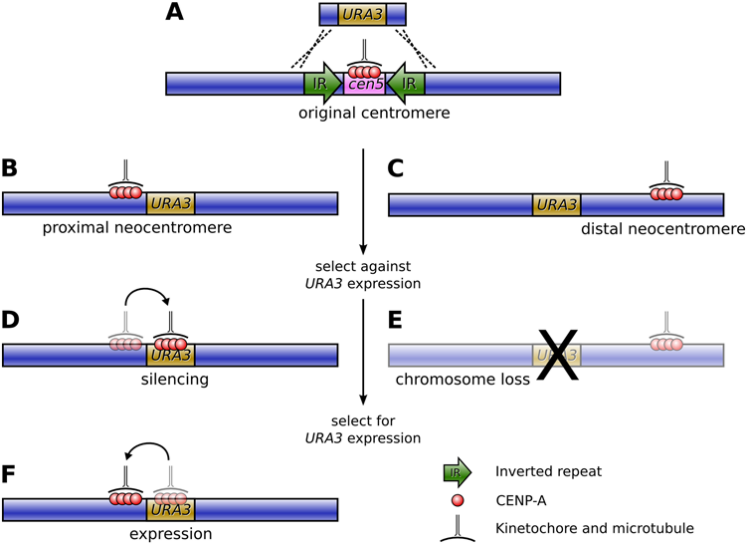
Formation of neocentromeres in *C. albicans*. (A) The existing centromere on Chromosome V, together with the surrounding inverted repeats, is replaced with the *URA3* gene via homologous recombination, resulting in neocentromere formation either proximal (B) or distal (C) to the original centromere. Selection against *URA3* expression results in either chromosome loss (E) or silencing of *URA3* through centromere shifting (D). If resistant colonies from the latter case are again grown on uridine-deficient media, a second shift in the position of the centromere restores *URA3* expression (F).

It was this question that Ketel et al., in this issue of *PLoS Genetics*, set out to answer [5]. The authors based their study on the pathogenic fungus *Candida albicans*, which has small, simple, regional centromeres flanked by inverted repeats, and extremely high rates of homologous recombination. Their approach was to specifically remove the centromeric DNA on Chromosome V by replacing it with *URA3*, a selectable marker gene, and observe the positioning and frequency of neocentromeres that resulted via chromatin immunoprecipitation for the fundamental centromere marker protein CENP-A (Figure 1A).

**Figure 2:**
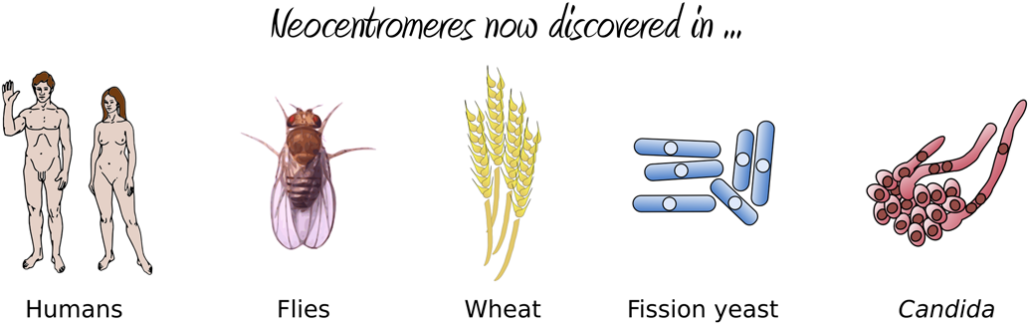
Organisms in which neocentromere formation has been reported. From left to right are: humans (reviewed in [4]), flies [6],[7], wheat [8], *Schizosaccharomyces pombe* [9], and *C. albicans* [5].

The results were striking: the authors found an extremely high frequency of neocentromere formation (with neocentromeres forming in all transformants) at multiple possible locations along Chromosome V. Essentially, these neocentromeres fell into two distinct classes: proximal neocentromeres, which formed close to the location of the original, excised centromere (Figure 1B); and distal neocentromeres, which formed at all other locations on the chromosome (Figure 1C). Although experimentally induced neocentromere formation has been previously investigated in flies [6],[7], plants [8], and other fungi [9], this is the first example, to our knowledge, where neocentromeres have been found to form at seemingly random chromosomal locations, similar to human neocentromeres (Figure 2).

In most cases, the size of the neocentromeres was similar to a normal *C. albicans* centromere, albeit with reduced quantities of CENP-A. Would the resulting neocentromeres be less stable during mitosis? To find out, Ketel et al. used a standard assay to gauge chromosome stability, growing the transformant strains on 5-FOA media, which is toxic to Ura+ cells. Those transformants with distal neocentromeres became resistant through loss of the neocentric chromosome at a rate comparable to control strains, suggesting that *Candida* neocentromeres suffered no loss of mitotic stability (Figure 1E).

However, transformants with proximal neocentromeres (near the selectable marker gene) became FOA-resistant at a much higher rate. Astonishingly, though, this was not due to higher rates of chromosome loss. In these strains the neocentromere had shifted onto the *URA3* gene, thereby silencing *URA3* expression (Figure 1D). Furthermore, moving the resistant strains back onto media selective for uridine synthesis resulted in the neocentromere shifting away from the gene and *URA3* expression being restored (Figure 1F).

Does this mean, then, that centromeres are incompatible with gene expression? Experiments such as the current work and recent reports in fission yeast [10]—where genes inserted within centromeric chromatin were similarly down-regulated—would suggest that this is the case. But these results are somewhat contradicted by results in human cells, where at both a neocentromere [11] and artificially generated chromosomes [12],[13] gene expression has been demonstrated despite the presence of CENP-A. Such observations may point to a different chromatin environment between humans and fungi at centromeres. Alternatively, it is possible that centromeric chromatin is merely impermissible to high levels of gene transcription—both experiments in fungi reported very low levels of reporter gene transcription still occurring. But such observations are intriguing considering recent reports of transcription at centromeres [14], and investigation of the precise relationship between centromeric chromatin and transcription is likely to become an important research focus in the future.

A key question regarding neocentromere formation has been whether there are any DNA sequence motifs required for a new centromere to arise. Using the three distal neocentromeres isolated in this study, Ketel et al. were unable to find any common sequence between the three regions. The only similarity, indeed, seemed to be that all neocentromeres formed within intergenic regions on the chromosome—not surprising, perhaps, considering the negative effect that centromeric chromatin appears to have on gene expression in *Candida*. It is unfortunate, though, that so few distal neocentromeres were analysed, making it impossible to tell if *C. albicans* has “hotspots” of neocentromere similar to those found on human chromosomes [4]. And what of the large number of proximal neocentromeres that arose? The high frequency of proximal neocentromere formation makes these neocentromeres difficult to explain through an occasional shifting or spreading of the centromeric signal. Perhaps there are other epigenetic marks conducive to centromere formation that lie outside of the excised *cen5* region.

So what can we conclude from this research? Clearly, *C. albicans* provides an excellent model system for studying the process of neocentromere formation, and the current work throws up many new questions regarding both the process of centromere formation and its impact upon transcription. What this work undeniably demonstrates, though, is that the ability to form neocentromeres is common from fungi to humans and is clearly an integral part of the genome.
